# Childhood disability and its associated perinatal characteristics in Bao’an district of Shenzhen, China

**DOI:** 10.1186/s12889-020-09623-3

**Published:** 2020-10-13

**Authors:** Xue Zhong, Xiaoli Zhao, Zhuoya Liu, Yuqin Guo, Liya Ma

**Affiliations:** Department of Child Healthcare, Shenzhen Baoan Women’s and Childiren’s Hospital, Jinan University, No.56 Yulv Road, Xin’an Street, Baoan District, Shenzhen City, 518100 Guangdong Province China

**Keywords:** Children, Disability, Mental disability, Perinatal factors

## Abstract

**Background:**

Disability has become a public health issues in China and around the world. This study aimed to report prevalence of children with disability by gender, delivery mode, birth weight, gestational age, birth defect and impairment type in the past 15 years in Baoan District, Shenzhen.

**Methods:**

Data of children with all types of disability from year 2004 to 2018 was obtained from the registry database of Baoan Disabled Persons Federation. Their perinatal information, including gender, delivery mode, birth weight, gestational age, birth defect were traced from Shenzhen Maternal and Child Health Management System and compared with the whole registered population live births information in this district. Data of children with disabilities were included to calculate the prevalence (%).

**Results:**

An overall prevalence of children disability was 1.02% in Baoan district, Shenzhen, in the past 15 years. The overall as well as mental disability prevalence rose from the first 5 years period (2004 to 2008) to the second 5 years of 2009 to 2013, and then dropped to the lowest level in the third 5 year of 2014–2018. Mental disability and intelligent disability accounted for the highest proportion. More than 70% of all kinds of the disabilities except mental disability were detected before 1 year old, 87% of the mental and intelligent disabilities were found before 3 years old. The Percentages of male, premature, low birth weight infants and children with cesarean, birth defect in disable children were higher than in whole population live birth children.

**Conclusions:**

The overall prevalence of disability declined in the district after specific policy engagement. Mental and intelligent disabilities were still the most common disability in the district. The age of 0–3 years was an important period for early childhood detection and intervention.

## Background

Disability is an umbrella term, covering impairments, activity limitations, and participation restrictions. An impairment is a problem in body function or structure; an activity limitation is a difficulty encountered by an individual in executing a task or action; while a participation restriction is a problem experienced by an individual in involvement in life situations [1]. According to World Report on Disability by WHO, about 15% of the world’s population lives with disability in 2011,at least 10% of the children were born with or acquired disability [2]. According to data of the second China national sample survey on disability in 2006 [3], there were an estimated 82.96 million disabled people in China this year, accounting for 6.34% of the total population. Among them, children aged 0–14 accounted for 4.66%, about 3.89 million in total. Disabled children face important challenges including undernutrition, abuse, more negative affected, victimization and lower belongingness [4–6]. Therefore disability has become a public health issues in China and around the world and also a great burden to family and the society. Families of a child with disability would need an additional cost to achieve the same living standards of families without a disabled child [7].

In recent years, Chinese government dedicates to improve the rehabilitation, education and employment of the disabled children. For example, Baoan district is the biggest district of Shenzhen covering 392.14 km^2^, with registered population of 572,900, the birth rate of 23.07‰. Since 2010, the local policy has been developed to improve the quality of life of disabled children as well as specific compensation for birth defects prevention and control, neonatal inherited metabolic disease screening, neonatal hearing screening, visual screening, follow-up of high-risk infants, early intervention of neurodevelopmental disorders, and rehabilitation assistance for disabled children, and etc.. People here come from all over the country with variety of cultural background, which could be a good representative to study childhood disability.

Since adverse perinatal conditions were related to increased risk of certain diseases during childhood [8–10], exploring the correlation between disability and perinatal characteristics might help to create public policies to reduce childhood disability. In this study, we use data in the past 15 years in Baoan District, Shenzhen, to report prevalence of children with disability by gender, delivery mode, birth weight, gestational age, birth defect and impairment type and try to explore the correlation between childhood disability and perinatal factors in order to provide evidence for government to take further measures. We believe the multiple policy supports, special multicultural backgrounds, powerful and convenient electronic database of Shenzhen city related to this study would be much helpful and useful to childhood disability studies and public health policy setting.

## Methods

### Data

Shenzhen Maternal and Child Health Management System (MCHMS) [11] is a continuously updated health care electronic database with an approximate 99.5% geographic coverage since its setting up in 2003. In addition to the mother’s demographic and pregnancy information, the perinatal and birth information of child including gender, delivery mode, gestational age, birth weight, Apgar score, birth defect would be uploaded to the MCHMS after every newborn’s birth by manual or automatically from electronic medical records in the hospital. The uploading rate and accuracy of birth information at each hospital will be monitored by the healthcare government in the district.

China Disabled Persons’ Federation (CDPF), founded in 1988, is an official agency for individuals with disability of different categories in China. A registry database is maintained by the CDPF’s district or county level office who registers persons with disability in that district or county. Individuals with disability who require government financial assistance or other services need an official disability certificate issued by CDPF in their district after evaluated by a certified physician. The CDPF organizes and carries out monitoring of the situation of disabled persons every year, including identity and list verification, follow-up investigation, collecting and analyzing data, implementing research subjects. The CDPF database includes basic information of each disable child, as well as identity information of child’s mother.

Data of children with all types of disability from year 2004 to 2018 in Baoan, Shenzhen was obtained from the registry database of CDPF. Their perinatal information, including gender, delivery mode, birth weight, gestational age, birth defect were traced from MCHMS and compared with the whole population live births information in the same period from MCHMS in this district. The two database were aligned based on the names of the child and his or her mother and the date of the child’s birth. To ensure the accuracy, birth weight and gestational age were checked to be consistent, which were registered in the database of both CDPF and MCHMS. The wrong information was corrected by contacting disabled child’s parents on phone. Prevalence of disability was calculated using data from the above two database. Data with missing information was deleted when studying each perinatal high risk factor.

### Disability evaluation and classification

Children with disability were evaluated and classified with the standards of China Classification and Grading Criteria of Disability by a certified physician in the hospitals accredited by CDPF. Disability was categorized into seven types including vision disability, hearing disability, speech disability, physical disability, intelligent disability, mental disability and multiple disability. In addition, mental disability included organic mental disorders, mental disorders due to psychoactive substance use, schizophrenia, schizotypal and delusional disorders, mood disorders, neurotic stress-related and somatoform disorders, behavior syndromes, disorders of adult personality and behavior, autism, and epilepsy [12]. Intelligent disability was defined by intelligence quotient score under 70, deficits in two or more adaptive behaviors [13].

### Statistical analysis

In this study, data of children with disabilities were included to calculate the prevalence(%).Specially, independent sample χ^2^ tests were performed to compare the status of disabilities and the whole population live births. All statistical analyses were carried out by the SPSS, version 25.0.*P* value < 0.05 was considered as statistically significant.

## Results

After linking the database of CDPF to the MCHMS, 636 disabled children with perinatal data were retrieved between 2004 and 2018. There were totally 62,516 registered live birth children in MCHMS during this period.

### Composition of the disabilities

**Fig. 1:**
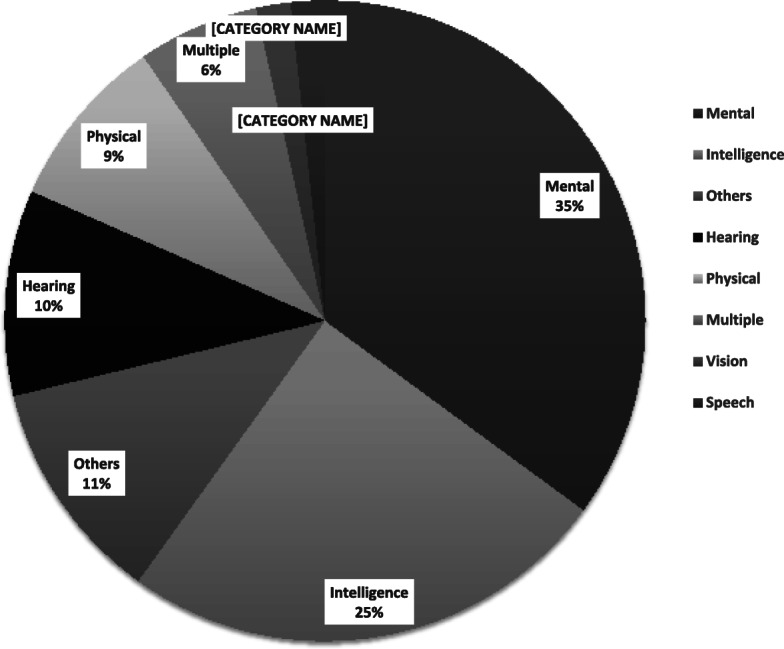
Childhood Disability Classification and Composition in Bao’an, Shenzhen

As presented in Fig. 1, among those disabled children, mental disability accounted for over one third (223 cases,35.06%),158 cases (24.84%) were intelligent disability, 66 cases (10.38%) were hearing disability, 56 cases (8.80%) were physical disability.

### Prevalence of the disabilities

**Fig. 2:**
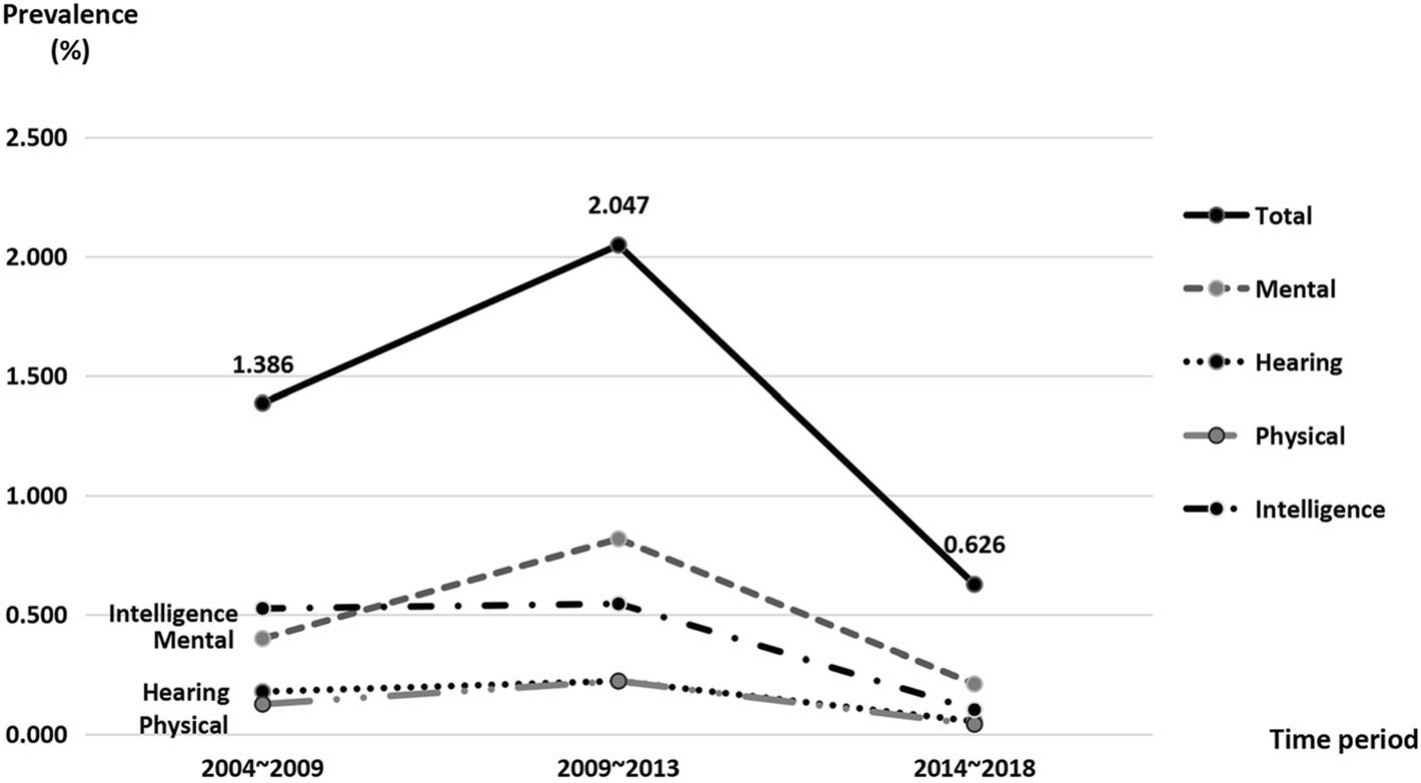
Prevalence of Children with Disability in Bao’an, Shenzhen

**Fig. 3:**
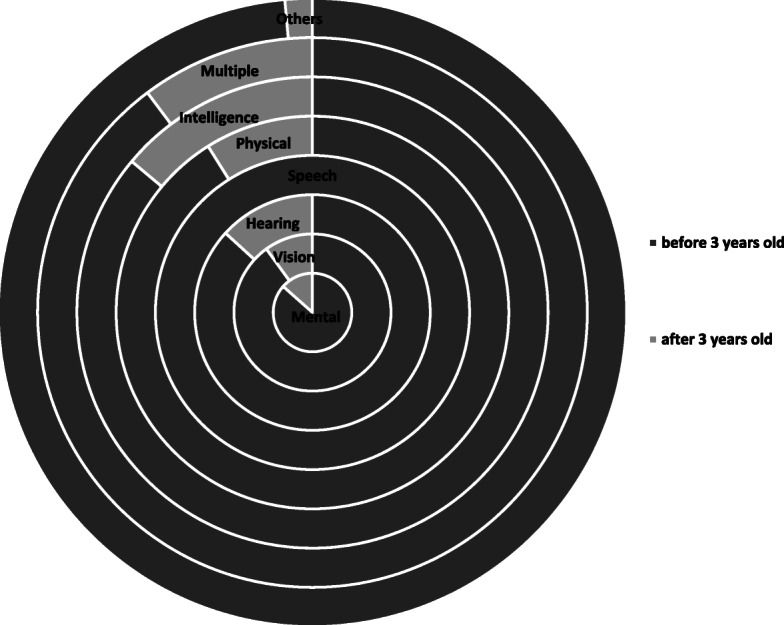
Age of Children with Disability in Bao’an, Shenzhen

The prevalence of overall and each disability of vision, hearing, speech, physical, intelligent, mental and multiple disability were shown in Table 1. The overall prevalence of children disability was 1.02% in Bao’an district, Shenzhen, in the past 15 years (636 cases among 62,516 live birth children). As shown in Table 1 and Fig. 2, the overall prevalence as well as mental disability rose from the first 5 years period (2004 to 2008) to the second 5 years of 2009 to 2013, then dropped to the lowest level in the third 5 year of 2014–2018. As shown in the Table 2 and Fig. 3, more than 70% of all kinds of the disabilities except mental disability were found before 1 year old, while 87% of the mental and intelligent disabilities were found before 3 years old.

**Table 1:** 5-Year Prevalence of Children with Disability in Bao’an, Shenzhen

**Time period**	**Perinatal infants**	**Mental**		**Hearing**		**Physical**		**Intelligence**		**Vision**	
		**n(%)**	**Prevalence** **(%)**	**n(%)**	**Prevalence** **(%)**	**n(%)**	**Prevalence** **(%)**	**n(%)**	**Prevalence** **(%)**	**n(%)**	**Prevalence** **(%)**
2004 ~ 2008	9525	38 (28.79)	0.40	17 (12.88)	0.18	12 (9.09)	0.13	50 (37.88)	0.52	7 (5.30)	0.07
2009 ~ 2013	12,114	99 (39.92)	0.82	27 (10.89)	0.22	27 (10.89)	0.22	66 (26.61)	0.54	3 (1.21)	0.02
2014 ~ 2018	40,877	86 (33.59)	0.21	22 (8.59)	0.05	17 (6.64)	0.04	42 (16.41)	0.10	1 (0.39)	0.00
Total	62,516	223 (35.06)	0.36	66 (10.38)	0.11	56 (8.81)	0.09	158 (24.84)	0.25	11 (1.73)	0.02
linear by linear		32.816		21.534		17.135		86.482		21.725	
*P*		0.000		0.000		0.000		0.000		0.000	
**Time period**	**Speech**			**Multiple**				**Others**		**Total**	
	**n(%)**	**Prevalence (%)**		**n(%)**		**Prevalence (%)**		**n(%)**	**Prevalence** **(%)**	**n(%)**	**Prevalence (%)**
2004 ~ 2008	0 (0)	0.00		5 (3.79)		0.05		3 (2.28)	0.03	132	1.39
2009 ~ 2013	3 (1.21)	0.02		20 (8.06)		0.17		3 (1.21)	0.02	248	2.05
2014 ~ 2018	8 (3.13)	0.02		14 (5.47)		0.03		66 (25.78)	0.16	256	0.63
Total	11 (1.73)	0.02		39 (6.13)		0.06		72 (11.32)	0.12	636	1.02
linear by linear	1.011			5.150				18.094		106.432	
*P*	0.375			0.000				0.000		0.000

**Table 2:** Age of Children with Disability in Bao’an, Shenzhen

Age (year)	Mental	Vision	Hearing	Speech	Physical	Intelligence	Multiple	Others	Total
	(n,%)	(n,%)	(n,%)	(n,%)	(n,%)	(n,%)	(n,%)	(n,%)	(n,%)
~ 1	104 (47)	8 (80)	48 (71)	9 (82)	44 (79)	108 (68)	32 (82)	62 (87)	415 (65)
~ 2	44 (20)	1 (10)	9 (13)	2 (18)	5 (9)	11 (7)	2 (5)	7 (10)	81 (13)
~ 3	45 (20)	0 (0)	2 (3)	0 (0)	2 (4)	17 (11)	1 (3)	1 (1)	68 (11)
~ 4	15 (7)	0 (0)	3 (4)	0 (0)	0 (0)	7 (4)	0 (0)	1 (1)	26 (4)
~ 5	5 (2)	0 (0)	2 (3)	0 (0)	1 (2)	2 (1)	0 (0)	0 (0)	10 (2)
~ 6	4 (2)	1 (10)	0 (0)	0 (0)	1 (2)	5 (3)	3 (8)	0 (0)	14 (2)
~ 14	6 (3)	0 (0)	4 (6)	0 (0)	3 (5)	8 (5)	1 (3)	0 (0)	22 (3)

### Perinatal characteristics of children with disability

The perinatal and birth information of children with disability were presented in Table 3 and compared with those of whole population live birth information.

**Table 3:** Birth Characteristics of Children with Disability in Bao’an, Shenzhe

Characteristics	Perinatal infants	Mental	Vision	Hearing	Speech	Physical	Intelligence	Multiple	Others	Total
	(n,%)	(n,%)	(n,%)	(n,%)	(n,%)	(n,%)	(n,%)	(n,%)	(n,%)	(n,%)
Gender										
Male	34,065 (54.49)	179 (80.27)	6 (54.55)	45 (67.16)	8 (72.73)	31 (55.36)	95 (60.13)	23 (58.97)	46 (64.79)	433 (68.08)
Female	28,451 (45.51)	44 (19.73)	5 (45.45)	22 (32.84)	3 (27.27)	25 (44.64)	63 (39.87)	16 (41.03)	25 (35.21)	203 (31.92)
χ^2^		59.568	0.000	4.336	1.475	0.017	2.019	0.316	3.033	46.925
*P*		0.000	0.997	0.037	0.225	0.896	0.155	0.574	0.082	0.000
Delivery mode										
Vaginal delivery	41,792 (66.85)	63 (42.00)	1 (25.00)	22 (57.89)	6 (75.00)	17 (43.59)	54 (48.21)	13 (48.15)	41 (61.19)	217 (48.76)
Cesarean	20,724 (33.15)	87 (58.00)	3 (75.00)	16 (42.11)	2 (25.00)	22 (56.41)	58 (51.79)	14 (51.85)	26 (38.81)	228 (51.24)
χ^2^		41.661	1.555	1.374	0.013	9.514	17.512	4.259	0.966	65.094
*P*		0.000	0.212	0.3	0.909	0.003	0.000	0.043	0.363	0.000
Birth weight (g)										
<2500	3132 (5.01)	17 (8.63	2 (28.57)	4 (6.45)	1 (9.09)	13 (26.00)	29 (19.21)	12 (32.43)	10 (16.13)	88 (15.25)
2500-	56,371 (90.17)	161 (81.73)	5 (71.43)	54 (87.10)	10 (90.91)	30 (60.00)	112 (74.17)	22 (59.46)	48 (77.42)	442 (76.60)
>4000	3013 (4.82)	19 (9.64)	0 (0.00)	4 (6.45)	0 (0.00)	7 (14.00)	10 (6.62)	3 (8.11)	4 (6.45)	47 (8.15)
χ^2^		15.971	5.480	0.598	0.920	33.201	65.451	29.575	11.052	141.965
*P*		0.000	0.085	0.742	0.681	0.000	0.000	0.000	0.004	0.000
Gestational age (week)										
<37	3670 (5.87)	12 (6.06)	2 (28.57)	3 (4.92)	2 (18.18)	16 (32.00)	21 (14.09)	14 (37.84)	9 (14.29)	79 (13.72)
≥ 37	58,846 (94.13)	186 (93.94)	5 (71.43)	58 (95.08)	9 (81.82)	34 (68.00)	128 (85.91)	23 (62.16)	54 (85.71)	497 (86.28)
χ^2^		0.013	3.064	0.002	3.016	56.901	18.135	61.533	6.606	62.844
*P*		0.910	0.080	0.965	0.133	0.000	0.000	0.000	0.010	0.000
Birth defect										
Yes	1413 (2.26)	8 (5.59)	1 (25.00)	3 (7.69)	2 (25.00)	2 (5.13)	14 (12.50)	5 (18.52)	11 (16.42)	46 (10.29)
No	61,103 (97.74)	143 (94.41)	3 (75.00)	36 (92.31)	6 (75.00)	37 (94.87)	98 (87.50)	22 (81.48)	56 (83.58)	401 (89.71)
χ^2^		4.977	9.356	5.198	18.700	1.450	48.588	32.195	60.757	130.123
*P*		0.012	0.087	0.058	0.013	0.220	0.000	0.000	0.000	0.000

More children with disability fell into the “male” group (68.08%) as compared to whole population live birth group (54.49%, *P* < 0.01). Simultaneously, obvious male predominance was noted in mental disabilities (80.27% in disable children vs. 54.49% in whole population, *P* < 0.01) and the hearing disabilities (67.16% vs. 54.49%,*P* < 0.05) .

Among 445 disabilities with information of delivery mode, the percentage of cesarean (51.24%) were higher than that of whole population live birth infants (33.15%), *P* < 0.01). Besides, the rate of cesarean in mental (58.00%), physical (56.41%) and intelligent (51.79%) were all higher than that of whole population live birth infants (*P* all < 0.01) .

The percentages of low birth weight (< 2500 g,15.25%)infants in disable children were 3 times as in whole population live birth children (5.01%), and fetal macrosomia (> 4000 g,8.15%) were nearly 2 times as in whole population live birth group (4.82%), while normal birth weight children (2500 g ~ 4000 g,76.60%) were far less than the percentages of the live birth group (90.17%), all with statistical significance (*P* < 0.01). Compared with normal birth weight, the percentages of low birth weight and fetal macrosomia were significantly higher than that of the live birth group (*P* < 0.01) in mental and physical disabilities. While in intelligent and multiple disabilities, proportion of low birth weight was more than that of the live birth group (*P* < 0.01).

The percentages of premature children in disable children (< 37 week, 13.72%) were twice as in the whole population live birth children (5.87%), and it was similar to physical, intelligent and multiple disability with statistical significance (*P* < 0.05) .

There were 447 disabilities had records with or without birth defect, among whom there were 46 disabilities with birth defect, including trisomy 21 syndrome, malformation and so on. The percentages of birth defect in disabilities (10.29%) were significantly higher than that in the whole population live birth group (2.26%), as well as mental, hearing, speech, intelligent and multiple disability, all with statistical significance (*P* < 0.05) .

## Discussion

Our study showed an overall prevalence of registered children disability was 1.02% in Baoan district, Shenzhen, China in the past 15 years, which was in keeping with the rates of 0.4 to 17% recorded overseas for earlier years [14–19] and slightly lower than data reported in China. For example, in a research with nationally representative data of 764,718 children aged 0–14 years in China, the prevalence of child disability dropped from 2.6 to 1.5% between 1987 and 2006 [20]. In a sampling survey of disability in 0–6 year-old children in China, the overall prevalence was 1.362% in 2006,0.155% for hearing disability, 0.160% for visual, 0.931% for intelligent, 0.424% for physical, and 0.101% for mental [21]. This might because Shenzhen is the Special Economic Zone of China, representing one of the most developed area in China.

The overall prevalence rose from the first 5 years period (2004 to 2009) to the second 5 years of 2009 to 2013, and then dropped to the lowest level. The rising prevalence of the first period was mainly because of the increase of mental disability, which is consistent with the rising trend of mental disability in the national surveys in 2006 [22]. In recent years, mental problems have been paid more and more attention. From 2006, child autism spectrum disorder (ASD), the most common type of mental disability in childhood, had formally included in disability survey and registered in CDPF. This might be the reason of rising prevalence in the first period. Globally, mental disorders has been the leading cause of disability in children and youth, and the proportion will increase [23]. The prevalence of ASD in China has also been increasing in these years along with the world [24]. Our study showed the most common type of childhood disability in Baoan, Shenzhen was mental disability, accounting for more than one third in all the types, with proportion of 35.06%.Since 2010 the government in this area has taken series of measures to reduce intelligent or other kinds of disabilities, which might be the reasons of declining of overall disability prevalence from the second period.

We found that nearly 90% disabled children were detected before 3 years old, with two thirds before 1 year old. As the main type of disabilities, intellectual disability is diagnosed primarily in infancy or the early childhood [25]. Besides, most parents noticed autism symptoms before age 3,and slower nonverbal cognitive or language growth was present at 12 months of age [26, 27]. This occurred to us there might be some risk factors in perinatal and early infancy to affect children disabilities. Thus we investigated birth and perinatal factors in the disable children and found that childhood disabilities might be correlated with birth or perinatal factors.

The significant correlation between gender and disability found in our study is keeping with other researches [15, 17, 28, 29]. Janet L. et al. found that male sex was more susceptible to adverse neonatal and poor neurological outcome in very preterm infants, maybe owing to the subtle aspects of brain injury [30]. Moreover, a study in U.S. population showed that cesarean delivery significantly contribute to ASD risk which was an important type of disabilities [31]. Cesarean delivery with general anesthesia might do harm to neurodevelopment in infant because of neurotoxicity [32]. Our results discovered the correlations between preterm birth and disability, is consistent with other research [33]. Maybe the impaired brain development related to preterm make the infants vulnerable to disabilities [34]. Moderate and late preterm birth was connected to smaller brain size, immature myelination of the internal capsule, and more undeveloped gyral folding, resulting in higher risk of neurodevelopmental disability with cognitive impairments at 2 years old [35]. Besides, the results of the current study appeared to agree with the opinion that low birth weight is at increased risk of children disability, probably suboptimal prenatal development might increase susceptibility to mental disorders [36]. Others also found that low birth weight children were at a high stake of neurodevelopmental and behavioral impairment, autism and pervasive developmental disorder [37, 38]. The possible link between birth defects and disability is also plausible. There are two kinds of birth defects: major and minor defects, the major defects is a malformation of an organ structure or function leading to disability or death, while the minor defect does not cause great damage to health [39, 40]. Many birth defects were connected with developmental disorders in childhood [41]. In a large population based study, Martha et al. found that intellectual performance of men with heart defects or cleft palate had adversely affected, probably related to ongoing complications by the defects and males with birth defects had six times the risk of disabilities, of which the degree change with the severity of the defects [42].

### Strengths and limitations

The strength of the study is that we used available data from CDPF to estimate the prevalence of childhood disability and its associated perinatal characteristics in Baoan district, Shenzhen, which is of great value for providing fundamental research on the contribution of early life factors to disabilities. In addition, compared with cross-sectional investigation at certain time point, our long-term longitudinal data could provide much richer information. However, there were some limitations in our study. The registration relies partly on the spontaneous reporting by parents, leading to missing of some handicapped children. Second, our study used the latest China Classification to define disability type, which was different from the international standard [43]. Third, the MCHMS does not cover all the perinatal factors that have harmful effects on disability, which also make it difficult to apply multiple regression to address the impact of confounders. Finally, although our study was based on registered population, who live relatively fixed, a few children born in Bao’an could register in CDPF of other areas and similarly children born from other areas could be registered in the CDPF of Bo’an district. Two-way flow might lead to a minor change of prevalence in the district.

## Conclusions

In conclusion, the overall prevalence of childhood disabilities declined in the district after policy changes. Mental and intelligent disabilities were the most common disability in the district, and the age of 0–3 years was an important period for early childhood detection and intervention. These findings are useful to support the decision of public policies which intended to promote integration of healthcare of children. More should be done to reduce the childhood disabilities from maternal and perinatal health care. Furthermore, the access available to health promotion, prevention, detection and early intervention of disabilities are still in need, as well as intensifying the specific public policies regarding recovery, education, jobs and social support. And further research is warranted on the contribution of early life factors to childhood disability.

## Data Availability

The datasets generated and/or analyzed during the current study are not publicly available due to the privacy of children with disabilities and their families, but are available from the corresponding author on reasonable request.

## Abbreviations

MCHMS: Maternal and Child Health Management System
CDPF: China Disabled Persons’ Federation
ASD: Autism spectrum disorder
